# Association between metabolically healthy obesity and kidney stones: results from the 2011–2018 National Health and Nutrition Examination Survey

**DOI:** 10.3389/fpubh.2023.1103393

**Published:** 2023-05-25

**Authors:** Weinan Chen, Sailimai Man, Yang Hong, Gaohaer Kadeerhan, Liang Chen, Qingquan Xu, Liulin Xiong, Tao Xu, Bo Wang, Xiaobo Huang

**Affiliations:** ^1^Department of Urology, Peking University People's Hospital, Beijing, China; ^2^Peking University Applied Lithotripsy Institute, Beijing, China; ^3^Department of Epidemiology and Biostatistics, School of Public Health, Peking University, Beijing, China; ^4^Meinian Institute of Health, Beijing, China; ^5^Peking University Health Science Center Meinian Public Health Institute, Beijing, China; ^6^National Cancer Center, National Clinical Research Center for Cancer, Cancer Hospital and Shenzhen Hospital, Chinese Academy of Medical Sciences and Peking Union Medical College, Shenzhen, China

**Keywords:** metabolically healthy obesity, precent body fat, insulin resistance, kidney stones, urolithiasis

## Abstract

**Introduction:**

The risk of kidney stones in metabolically healthy obesity (MHO) individuals is largely unexplored. This study using percent body fat (%BF) to categorize obesity, to investigate the association between MHO as well as other metabolic syndrome-obesity combined phenotypes and kidney stones in a national representative population.

**Materials and methods:**

This cross-sectional study included 4,287 participants in the National Health and Nutrition Examination Survey from 2011 to 2018. Metabolically healthy status was defined as not having any component of metabolic syndrome or insulin resistance. Obesity was identified by %BF, which was measured and assessed by dual-energy x-ray absorptiometry (DXA) scan. Participants were cross-classified by metabolic health and obesity status. The outcome was self-report kidney stones. Multivariable logistic regression model was used to examine the association between MHO and kidney stones.

**Results:**

A total of 358 participants had kidney stones [weighted prevalence (SE): 8.61% (0.56%)]. The weighted prevalence (SE) of kidney stones in MHN, MHOW, and MHO groups was 3.13% (1.10%), 4.97% (1.36%), and 8.55% (2.09%), respectively. After adjusting for age, sex, race and ethnicity, education level, smoking status, alcohol consumption, physical activity, daily water intake, CKD stage 3–5, and hyperuricemia, MHO individuals (OR: 2.90, 95% CI: 1.18, 7.0) had a significantly higher risk of kidney stones than those with metabolically healthy normal weight. In metabolically healthy participants, a 5% increment in %BF was associated with a significantly higher risk of kidney stones (OR: 1.60, 95% CI: 1.20, 2.14). Furthermore, a nonlinear dose–response relationship between %BF and the kidney stones was observed in metabolically healthy participants (*P* for non-linearity = 0.046).

**Conclusion:**

Using %BF to define obesity, MHO phenotype was significantly associated with higher risks of kidney stones, suggesting that obesity can independently contribute to kidney stones in the absence of metabolic abnormalities and insulin resistance. Regarding kidney stones prevention, MHO individuals might still benefit from lifestyle interventions aimed at healthy body composition maintenance.

## Introduction

Kidney stones disease is a common problem that affects up to approximately 15% of the world population (1). In the past several decades, the prevalence of kidney stones disease in almost all countries has seemed to be increasing continuously due to the rise in the prevalence of risk factors for kidney stones (2). Obesity and metabolic syndrome have been found to be risk factors for kidney stones (3, 4). In a large proportion of individuals who suffered from kidney stones disease, the deleterious effect of obesity on stone formation is often mediated by adiposity-related metabolic syndrome (4). However, a subset of individuals with obesity does not present metabolic syndrome, i.e., metabolically healthy obesity (MHO) (5). These individuals may display distinct disease outcomes compared with both metabolically unhealthy obesity (MUO) phenotype and metabolically healthy normal weight (MHN) phenotype (6). Emerging evidence is reaching the consensus that the MHO phenotype is not a relatively benign condition in terms of urological disorders, and bladder cancer (7, 8). However, studies on the association between MHO and kidney stones are limited, and the assessment of MHO in previous studies was mainly based on body mass index (BMI) (9, 10), which might bias the results since it was believed that BMI was hard to accurately differentiate between adipose and non-adipose tissues (11). Furthermore, available studies on the association between MHO and kidney stones mostly come from less representative samples (9, 10). Hence, studies based on a nationally representative population, and using relatively accurate indicators to categorize obesity could provide reliable evidence on the association between metabolic syndrome-obesity combined phenotypes and kidney stones, thereby helping to identify high-risk groups and promote kidney stones prevention.

In the National Health and Nutrition Examination Survey (NHANES), which was conducted in a representative sample of US population (12), dual-energy x-ray absorptiometry (DXA) scans were used to assess the body fat parameters of the participants. Therefore, we draw data from the NHANES, using percent body fat (%BF) to categorize obesity status, to investigate the association between metabolic syndrome-obesity combined phenotypes and kidney stones.

## Materials and methods

### Study population

**Figure 1:**
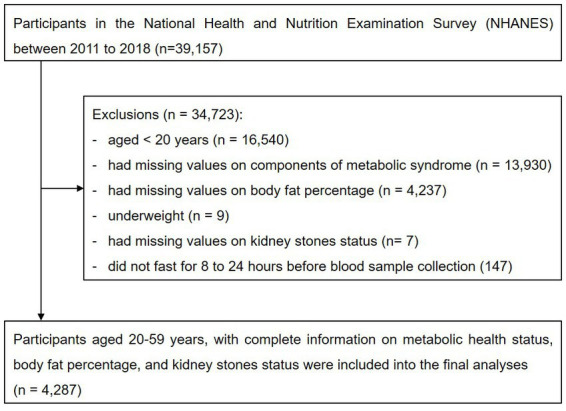
Flowchart of the study participants.

This was a cross-sectional study using data from the four consecutive cycles of NHANES from 2011 to 2018, which was conducted in the US by the National Center for Health Statistics of the Centers for Disease Control and Prevention. NHANES is a cross-sectional survey program conducted every 2 years, aimed at including individuals who are representative of the general, noninstitutionalized population of all age groups. A stratified, multistage, clustered probability sampling design is applied during the survey. The survey consists of a structured interview conducted in the home, followed by a standardized health examination that includes a physical examination and laboratory tests. Full methodology of the data collection process is available elsewhere (12). Initially, we included participants enrolled in the four consecutive study cycles from 2011 to 2018 (*n* = 39,157). Participants whose age < 20 years (*n* = 16,540), had missing values on components of metabolic syndrome (*n* = 13,930), did not undergone DXA scan examination (*n* = 4,273), were underweight (*n* = 9), had missing values on kidney stones status (*n* = 7), or did not fast for 8–24 h before blood sample collection (*n* = 147) were excluded from the study. Finally, 4,287 participants were included in the analyses (Figure 1).

The original survey was approved by the Centers for Disease Control and Prevention Research Ethics Review Board and written informed consent was obtained from all adult participants. The present analyses were approved by the Institutional Review Board of Peking university People’s Hospital (approval ID: 2020PHB 125–01).

### Data collection

Data on demographic characteristics, educational level, smoking status, alcohol consumption, physical activities, and dietary intake were collected using standardized questionnaires. Height, weight, waist circumference, and systolic and diastolic blood pressure were measured using calibrated instruments with standard protocols during the mobile examination center visit. Fasting venous blood samples were collected in a subsample of the total participants in the NHANES. Laboratory methods of measuring fasting blood glucose, insulin, total cholesterol, triglycerides, low-density lipoprotein cholesterol, high-density lipoprotein cholesterol, serum creatinine, uric acid, blood urea nitrogen, alkaline phosphatase, aspartate aminotransferase (AST), alanine aminotransferase (ALT), and albumin are reported in detail elsewhere (13).

The whole-body DXA scans were conducted in the four study cycles using a Hologic QDR 4500A fan-beam densitometer (Hologic, Inc., Bedford, MA, USA) following the manufacturer’s protocol (14). The principle of whole-body DXA scan relies on the different X-ray absorptions of tissues with different densities (e.g., bone vs. soft tissue) to determine the type and characteristics of the tissues. The accuracy of using DXA to measure body fat has been verified as good or very good by previous studies (15, 16). Each DXA scan was reviewed and analyzed by the Department of Radiology of University of California, San Francisco, using standard radiologic techniques and study-specific protocols developed for the NHANES. Hologic Discovery software version 12.1 was used to analyze DXA examinations and provide body composition data. %BF was determined as a ratio of fat mass over total body mass (including bone mineral mass). In the present study, %BF were extracted from the DXA scan data for each participant to conduct the analyses.

### Assessment of obesity-metabolic syndrome phenotypes

Participants were classified as meeting criteria for normal weight, overweight, or obesity according to %BF based on the criteria recommended by Bray (Supplementary Table S1) (17). Metabolic health was defined based on the 2009 harmonized criteria of metabolic syndrome and homeostasis model assessment of insulin resistance (HOMA-IR) (18, 19). Participants who had none of the following five components of metabolic syndrome were considered metabolically healthy: (1) systolic blood pressure ≥ 130 mmHg or diastolic blood pressure ≥ 85 mmHg or self-reported hypertension or taking antihypertensive medication; (2) fasting blood glucose ≥ 5.6 mmol/L or self-reported diabetes or using antidiabetic drugs; (3) high-density lipoprotein cholesterol <1.03 mmol/L for men and < 1.29 mmol/L for women or using lipid-lowering medication; (4) triglycerides ≥1.7 mmol/L or using lipid-lowering medication; and (5) HOMA-IR ≥ 2.5 (calculated by insulin*glucose/22.5). Those who had ≥1 component were categorized as metabolically unhealthy. Finally, participants were categorized into six phenotypes: MHN, metabolically healthy overweight (MHOW), MHO, metabolically unhealthy normal weight (MUN), metabolically unhealthy overweight (MUOW), and MUO. To comprehensively explore the association of obesity with kidney stones, we further categorized participants into different groups using BMI and waist circumference (WC) criteria. BMI was calculated as body weight in kilograms divided by the square of height in meters. For BMI criteria, normal weight (BMI 18.5–24.9 kg/m^2^), overweight (BMI 25.0–29.9 kg/m^2^), and obese (BMI ≥ 30.0 kg/m^2^) were categorized according to the cut-offs suggested by the World Health Organization (20). For WC criteria, participants were also categorized into three WC groups corresponding to the normal weight, overweight, and obese groups in BMI category. According to the recommendation of the World Health Organization, WC ≥ 80 cm for females and WC ≥ 94 cm for males (WC ≥ 90 for Asian American males) were the cut-off points for central obesity (18); moreover, the median values of WC among females (75.3 cm) and males (85.8 cm for others or 83 cm for Asian American males) without central obesity in this study were used as the cut-offs between normal and medium WC.

### Assessment of kidney stones status

Questions on kidney conditions, including kidney stones status, were asked in the home, by trained interviewers, using the Computer-Assisted Personal Interview (CAPI) system. Participants reported their status of kidney stones by responding to the question: “Have you ever had kidney stones?” Participants who answered “Yes” were defined as having kidney stones (21).

### Assessment of covariates

Sex was categorized as male or female. Age was analyzed as continuous variable. Race and ethnicity were categorized as non-Hispanic white, non-Hispanic black, Hispanic, Asian, and others. Educational level was categorized as less than 12 grade, high school, college, or above. Smoking status was categorized into never, former, and current smokers. Alcohol consumption was categorized into not excess, and excess (≥ 30 g/d for male, ≥ 20 g/d for female). Physical activity was classified as inactive (no moderate-to-vigorous physical activity), and active (any moderate-to-vigorous physical activity during a typical week) groups. Daily water intake was categorized into three groups according to the 33.33 and 66.67% percentiles. Estimated glomerular filtration rate (eGFR) was calculated according to the Chronic Kidney Disease Epidemiology Collaboration equation (22), and chronic kidney disease (CKD) stage 3–5 was defined as an estimated glomerular filtration rate < 60 mL/min/1.73 m^2^ (23). Hyperuricemia was defined as uric acid ≥ 420 μmol/L. Serum analytes, including lipid profile, serum creatine, uric acid, etc., were analyzed as continuous variables. Dietary factors, including protein, carbohydrate, fiber, and total fat were analyzed as continuous variables.

### Statistical analyses

Given the data collected in the NHANES were obtained through a stratified, multistage probability sample design, we used survey analyses procedures to account for sample weights, stratification, and clustering in our study. Sample size and characteristics were described according to different metabolic health-obesity phenotypes. Data were presented as weighted proportions (SE) for categorical variables and as weighted means ± SE for continuous variables. Logistic regression models were used to calculate the odds ratios (ORs) and 95% confidence intervals (CIs). Model 1 was not adjusted. Model 2 was adjusted for age, sex, and race and ethnicity. Model 3 was adjusted for age, sex, race and ethics, education level, smoking status, alcohol consumption, physical activity, daily water intake, CKD stage 3–5, and hyperuricemia. The covariates adjusted in the model were risk factors of kidney stones that were well-established in previous studies (1, 2, 24–27). Trend tests were conducted by treating the metabolically healthy or unhealthy phenotypes as continuous variables, which were generated by assigning a median %BF/BMI/WC value to each phenotype. Restricted cubic splines with knots at the 5^th^, 27.5^th^, 50th, 72.5^th^, and 95^th^ percentiles were used to further explore the shape of the dose–response relationship between %BF and kidney stones in metabolically healthy and unhealthy participants, respectively. The multivariable-adjusted model was stratified by demographic and lifestyle characteristics as potential modifiers: age (<40 or ≥40 years), sex (male or female), smoking status (current or none-current), and physical activity (inactive or active). The multiplicative interaction terms between these subgroups and metabolic health-obesity phenotypes were added to the fully adjusted model, and models with and without multiplicative interaction terms were compared using the likelihood ratio test. The following sensitivity analyses were conducted to examine the robustness of our findings: (1) additionally adjusting systolic blood pressure, diastolic blood pressure, glucose, triglycerides, high-density lipoprotein-cholesterol, and HOMA-IR (all were analyzed as continuous variables) in the multivariate logistic regression model and (2) additionally adjusting dietary protein intake, dietary carbohydrate intake, dietary fiber intake, and dietary intake of total fat (all were analyzed as continuous variables) in the multivariate logistic regression model.

All statistical analyses were performed using R version 3.6.2. The “survey” package in R was used to account for the survey design analyses. The “rms” package in R was used to conduct logistic regression analyses. The “rms” and “ggplot2” packages were used to perform restricted cubic spline analyses. All *p-*values were two-sided, and statistical significance was defined as *p* < 0.05.

## Results

### Characteristics of participants cross-classified by metabolic health and obesity

The characteristics of the study participants across metabolic health-obesity phenotypes defined by %BF are presented in Table 1. Among the 4,287 study participants, the mean (SE) age was 39.16 (0.27) years, and 50.53% were male. Metabolically healthy phenotypes accounted for 26.45% of the total study population. Among metabolically healthy participants, 277 (24.43%) participants were defined as having the MHO phenotype. Compared with the MUO phenotype, individuals with MHO had a favorable metabolic profile. On the contrary, compared with individuals with the MHN phenotype, MHO individuals were more likely to have fewer healthy metabolic parameters, CKD stage 3–5, and hyperuricemia.

**Table 1:** Characteristics of the study participants according to metabolic health-obesity (defined by %BF) phenotypes.

Characteristics	Overall	MHN	MHOW	MHO	MUN	MUOW	MUO
No. of participants	4,287	445	412	277	399	1,023	1731
Age, year^†^	39.16 ± 0.27	33.26 ± 0.73	35.64 ± 0.70	34.54 ± 0.74	41.25 ± 0.88	42.55 ± 0.45	40.08 ± 0.36
Male (%)^‡^	50.53 (1.02)	36.84 (3.43)	37.53 (3.24)	35.31 (3.29)	55.49 (3.05)	58.90 (2.15)	54.29 (1.48)
Hispanic (%)^‡^	18.39 (1.60)	9.49 (1.53)	18.30 (2.90)	18.54 (2.98)	11.00 (1.68)	18.24 (1.90)	22.71 (1.98)
College education (%)^‡^	65.19 (1.79)	77.10 (2.63)	74.00 (3.41)	71.86 (3.68)	62.50 (3.36)	60.87 (3.22)	61.54 (1.76)
Current smokers (%)^‡^	21.83 (1.14)	18.17 (2.42)	15.36 (2.02)	16.46 (2.68)	34.25 (3.67)	25.04 (2.27)	20.75 (1.09)
Excess alcohol consumption (%)^‡^	16.58 (0.74)	20.91 (3.08)	14.28 (1.77)	17.35 (2.97)	23.77 (3.80)	17.72 (1.82)	13.48 (1.13)
Inactive (%)^‡^	51.57 (1.05)	56.35 (3.18)	58.55 (3.32)	55.73 (3.68)	44.07 (3.20)	50.96 (2.16)	49.77 (1.62)
Daily water intake, L/day^†^	2.59 (0.06)	2.94 (0.20)	2.58 (0.14)	2.78 (0.23)	2.50 (0.16)	2.49 (0.11)	2.53 (0.10)
%BF, %^†^	32.90 ± 0.22	25.26 ± 0.40	32.04 ± 0.45	38.49 ± 0.48	24.28 ± 0.46	30.37 ± 0.28	37.81 ± 0.22
BMI, kg/m^2†^	28.62 ± 0.17	21.71 ± 0.17	24.77 ± 0.18	29.82 ± 0.43	23.31 ± 0.21	27.18 ± 0.15	33.40 ± 0.23
WC, cm^†^	97.21 ± 0.42	78.27 ± 0.48	87.23 ± 0.48	98.21 ± 0.99	83.95 ± 0.62	94.82 ± 0.42	109.38 ± 0.45
SBP, mmHg^†^	118.05 ± 0.33	109.05 ± 0.59	109.94 ± 0.49	110.95 ± 0.71	120.33 ± 0.93	121.62 ± 0.65	121.28 ± 0.40
DBP, mmHg^†^	71.03 ± 0.26	65.06 ± 0.53	66.47 ± 0.55	66.58 ± 0.61	70.50 ± 0.73	73.15 ± 0.46	73.52 ± 0.37
FBG, mmol/L^†^	5.78 ± 0.03	5.05 ± 0.02	5.10 ± 0.02	5.08 ± 0.02	5.80 ± 0.10	6.02 ± 0.07	6.13 ± 0.05
Insulin, uU/mL^†^	11.88 ± 0.34	4.76 ± 0.15	6.16 ± 0.15	7.05 ± 0.16	7.44 ± 0.39	11.47 ± 0.37	17.43 ± 0.60
HOMA-IR^†^	3.27 ± 0.12	1.07 ± 0.03	1.40 ± 0.04	1.60 ± 0.04	2.00 ± 0.12	3.12 ± 0.12	5.03 ± 0.22
TC, mmol/L^†^	4.91 ± 0.02	4.55 ± 0.05	4.64 ± 0.05	4.82 ± 0.06	4.84 ± 0.08	5.12 ± 0.04	4.98 ± 0.04
TG, mmol/L^†^	1.33 ± 0.02	0.71 ± 0.02	0.82 ± 0.02	0.89 ± 0.03	1.23 ± 0.07	1.59 ± 0.06	1.58 ± 0.04
HDL-C, mmol/L^†^	1.38 ± 0.01	1.69 ± 0.03	1.58 ± 0.02	1.47 ± 0.02	1.49 ± 0.02	1.30 ± 0.02	1.24 ± 0.01
LDL-C, mmol/L^†^	2.94 ± 0.02	2.54 ± 0.04	2.68 ± 0.04	2.94 ± 0.05	2.81 ± 0.07	3.10 ± 0.03	3.05 ± 0.03
Scr, μmol/L^†^	74.42 ± 0.45	73.44 ± 1.07	71.81 ± 0.98	71.62 ± 1.21	78.68 ± 1.67	76.64 ± 0.72	73.57 ± 0.65
UA, μmol/L^†^	317.97 ± 1.71	276.07 ± 4.80	286.58 ± 4.86	302.13 ± 5.57	298.24 ± 4.83	324.28 ± 3.71	341.33 ± 1.96
BUN, mmol/L^†^	4.54 ± 0.03	4.51 ± 0.10	4.39 ± 0.08	4.22 ± 0.07	4.62 ± 0.11	4.68 ± 0.07	4.55 ± 0.04
eGFR, mL/min/1.73m^2†^	102.99 ± 0.50	105.74 ± 1.09	105.13 ± 1.23	106.20 ± 1.49	99.94 ± 1.15	99.23 ± 0.75	104.01 ± 0.51
AST, U/L^†^	24.91 ± 0.33	22.91 ± 0.59	22.61 ± 0.48	22.35 ± 0.86	26.71 ± 1.50	25.60 ± 0.72	25.68 ± 0.64
ALT, U/L^†^	25.72 ± 0.32	19.33 ± 0.70	20.01 ± 0.63	21.41 ± 1.21	24.70 ± 1.76	26.63 ± 0.76	29.43 ± 0.55
Alb, g/dL^†^	4.29 ± 0.01	4.39 ± 0.02	4.36 ± 0.02	4.24 ± 0.02	4.35 ± 0.02	4.32 ± 0.02	4.21 ± 0.01
CKD stage 3–5 (%)^‡^	0.92 (0.17)	0.35 (0.21)	0.00 (0.00)	0.55 (0.32)	2.61 (1.38)	1.20 (0.41)	0.85 (0.19)
Hyperuricemia (%)^‡^	10.45 (0.56)	2.74 (1.00)	4.56 (1.28)	6.36 (1.91)	5.58 (1.59)	12.94 (1.42)	14.47 (0.92)
Dietary protein (gm)^†^	86.35 ± 0.87	87.30 ± 2.92	82.79 ± 2.75	75.05 ± 2.66	93.59 ± 3.35	88.49 ± 1.83	86.02 ± 1.31
Dietary carbohydrate (gm)^†^	264.38 ± 2.54	264.15 ± 8.45	244.40 ± 7.76	235.47 ± 7.21	291.12 ± 8.55	275.21 ± 6.05	262.03 ± 3.73
Dietary fiber (gm)^†^	17.32 ± 0.26	19.38 ± 0.65	17.12 ± 0.70	16.33 ± 0.75	17.87 ± 0.67	17.72 ± 0.48	16.59 ± 0.35
Dietary total fat (gm)^†^	87.01 ± 0.97	87.57 ± 2.94	80.92 ± 2.75	80.42 ± 2.63	90.70 ± 3.38	88.72 ± 2.00	87.66 ± 1.54
Kidney stones (%)^‡^	8.61 (0.56)	3.13 (1.10)	4.97 (1.36)	8.55 (2.09)	6.81 (1.47)	9.55 (1.14)	10.98 (0.82)

%BF, percent body fat; BMI, body mass index; WC, waist circumference; SBP, systolic blood pressure; DBP, diastolic blood pressure; FBG, fasting blood glucose; HOMA-IR, homeostasis model assessment of insulin resistance; TC, total cholesterol; TG, triglycerides; HDL-C, HDL cholesterol; LDL-C, LDL cholesterol; Scr, serum creatinine; UA, uric acid; BUN, blood urea nitrogen; eGFR, estimated glomerular filtration rate; AST, aspartate aminotransferase; ALT, alanine aminotransferase; Alb, albumin; CKD, chronic kidney disease; MHN, metabolically healthy normal weight; MHOW, metabolically healthy overweight; MHO, metabolically healthy obesity; MUN, metabolically unhealthy normal weight; MUOW, metabolically unhealthy overweight; MUO, metabolically unhealthy obesity. ^†^Data are presented as the weighted mean ± SE. ^‡^Data are presented as the weighted prevalence (SE).

A total of 358 participants had kidney stones [weighted prevalence (SE): 8.61% (0.56%)]. The weighted prevalence (SE) of kidney stones in MHN, MHOW, and MHO groups was 3.13% (1.10%), 4.97% (1.36%), and 8.55% (2.09%), while the figures were relatively higher in metabolically unhealthy groups, with the weighted prevalence (SE) increased to 6.81% (1.47%), 9.55% (1.14%), and 10.98% (0.82%) in MUN, MUOW, and MUO groups (Table 1).

### Associations between metabolic health-obesity phenotypes and kidney stones

The association between kidney stones and metabolic health-obesity phenotypes (defined by %BF) is presented in Table 2. After adjusting for age, sex, race, education level, smoking status, alcohol consumption, physical activity, daily water intake, CKD stage 3–5, and hyperuricemia, individuals with MHO (OR: 2.90, 95% CI: 1.18, 7.08) had a significantly higher risk of kidney stones than those with MHN. The corresponding OR (95% CI) for MHOW was 1.62 (0.56–4.05). Individuals with MUO had the highest OR (95% CI) among all phenotypes (OR: 3.82, 95% CI: 1.79, 8.14). In metabolically healthy participants, a 5% increment in %BF was associated with a significantly higher risk of kidney stones (OR: 1.60, 95% CI: 1.20, 2.14), while the corresponding OR (95%CI) for metabolically unhealthy participants was 1.17 (1.00–1.37). Significant trend associations were observed for %BF and kidney stones in metabolically healthy and unhealthy participants (All *P* for trend <0.05).

**Table 2:** The association between metabolic health-obesity phenotypes (defined by %BF) and kidney stones.

Metabolic health-obesity phenotypes	Events	Participants	Model 1^†^ OR (95% CI)	Model 2^‡^ OR (95% CI)	Model 3^§^ OR (95% CI)
Metabolically healthy participants					
MHN	12	445	Reference	Reference	Reference
MHOW	22	412	1.62 (0.56–4.65)	1.56 (0.53–4.59)	1.51 (0.51–4.53)
MHO	18	277	2.90 (1.18–7.08)	2.98 (1.20–7.42)	2.92 (1.15–7.42)
Per 5% increment in %BF			1.33 (1.07–1.66)	1.56 (1.17–2.07)	1.60 (1.20–2.14)
P for trend			0.026	0.026	0.023
Metabolically unhealthy participants					
MUN	25	399	2.26 (0.94–5.44)	2.08 (0.83–5.23)	1.99 (0.77–5.14)
MUOW	95	1,023	3.27 (1.49–7.17)	2.91 (1.26–6.72)	2.78 (1.19–6.50)
MUO	186	1731	3.82 (1.79–8.14)	3.62 (1.64–7.97)	3.44 (1.55–7.63)
Per 5% increment in %BF			1.16 (1.08–1.26)	1.19 (1.02–1.39)	1.17 (1.00–1.37)
P for trend			0.008	0.005	0.007

%BF, percent body fat; MHN, metabolically healthy normal weight; MHOW, metabolically healthy overweight; MHO, metabolically healthy obesity; MUN, metabolically unhealthy normal weight; MUOW, metabolically unhealthy overweight; MUO, metabolically unhealthy obesity; OR, odds ratio; CI, confidence interval. ^†^Model 1 was not adjusted. ^‡^Model 2 was adjusted for age, sex, and race and ethics. ^§^Model 3 was adjusted for age, sex, race and ethics, education level, smoking status, alcohol consumption, physical activity, daily water intake, CKD stage 3–5, and hyperuricemia.

**Figure 2:**
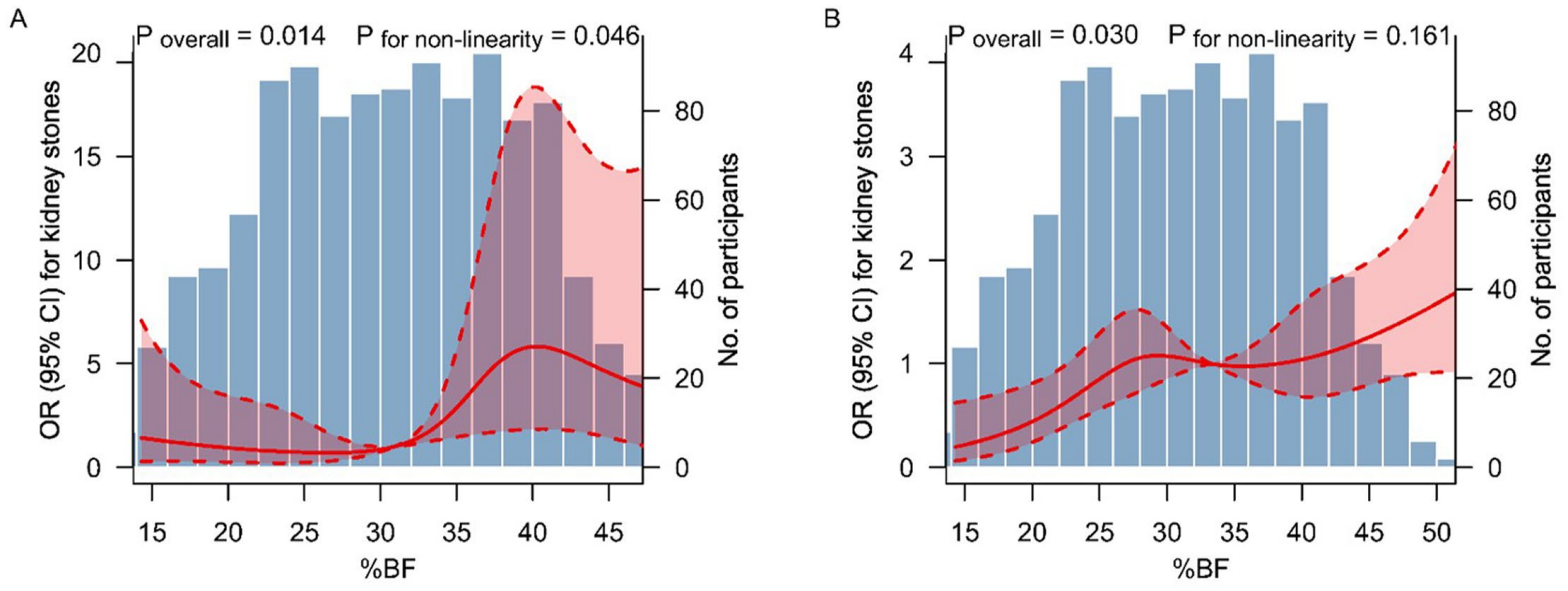
Dose–response relationship between %BF level and kidney stones. The association between %BF level and kidney stones among metabolically healthy **(A)** and unhealthy **(B)** participants. The solid lines and shaded areas represent the ORs and corresponding 95% CIs. The models were adjusted for age, sex, race and ethics, education level, smoking status, alcohol consumption, physical activity, daily water intake, CKD stage 3–5, and hyperuricemia. *p-*values for non-linearity were obtained using a chi-squared test to compare nested models. %BF, percent body fat; OR, odds ratio; CI, confidence interval.

Figure 2 shows the results of restricted cubic spline analyses. A non-linear dose–response relationship between %BF and the kidney stones was observed in metabolically healthy participants (*P* for non-linearity = 0.046) (Figure 2A). However, the dose–response relationship was approximately linear throughout the range of its levels in metabolically unhealthy participants (*P* for non-linearity = 0.161) (Figure 2B).

The results were largely unchanged when using the BMI or WC criteria to categorize various obesity phenotypes (Supplementary Table S2). When using BMI to define obesity, the multivariate adjusted ORs (95% CIs) comparing individuals with MHOW, MHO, MUN, MUOW, and MUO to MHN were 2.20 (1.13–4.32), 2.89 (1.01–8.24), 1.72 (0.86–3.44), 2.72 (1.54–4.82), and 3.70 (2.06–6.64), respectively. The corresponding ORs (95% CIs) when using WC categories exhibited a similar pattern.

Subgroup analyses are presented in Table 3. No significant interactions of metabolic health-obesity phenotypes with sex, age, smoking status, and physical activity on the risk of kidney stones were detected (all *P* for interaction >0.05).

**Table 3:** Subgroup analyses of the association between metabolic health-obesity (defined by %BF) phenotypes and kidney stones.

Subgroup^†^	MHN OR (95% CI)	MHOW OR (95% CI)	MHO OR (95% CI)	MUN OR (95% CI)	MUOW OR (95% CI)	MUO OR (95% CI)	P for interaction
Sex							0.395
Male	Reference	0.45 (0.07–2.92)	1.35 (0.30–6.13)	0.54 (0.17–1.70)	1.19 (0.39–3.63)	1.63 (0.56–4.79)	
Female	Reference	3.18 (0.75–13.39)	4.87 (1.34–17.69)	5.16 (1.28–20.83)	5.29 (1.48–18.93)	6.35 (1.94–20.75)	
Age							0.082
<40 years	Reference	2.94 (0.79–10.86)	4.32 (1.23–15.13)	5.18 (1.41–18.94)	4.88 (1.59–14.97)	5.57 (1.78–17.44)	
≥40 years	Reference	0.18 (0.03–1.13)	1.50 (0.25–9.12)	0.27 (0.05–1.53)	0.86 (0.24–3.03)	1.28 (0.36–4.52)	
Smoking status							0.947
Current smokers	Reference	1.16 (0.06–23.13)	2.48 (0.31–19.64)	1.61 (0.21–12.52)	2.21 (0.32–15.46)	3.12 (0.46–21.11)	
Non-current smokers	Reference	1.57 (0.51–4.87)	3.02 (1.06–8.64)	2.22 (0.69–7.17)	2.90 (1.14–7.41)	3.51 (1.48–8.33)	
Physical activity status							0.271
Active	Reference	2.12 (0.10–11.22)	3.89 (0.95–15.88)	3.22 (0.71–14.60)	2.88 (0.74–11.22)	3.31 (0.87–12.52)	
Inactive	Reference	1.13 (0.31–4.20)	2.37 (0.72–7.86)	0.87 (0.24–3.21)	2.65 (0.92–7.64)	3.68 (1.29–10.50)	

%BF, percent body fat; MHN, metabolically healthy normal weight; MHOW, metabolically healthy overweight; MHO, metabolically healthy obesity; MUN, metabolically unhealthy normal weight; MUOW, metabolically unhealthy overweight; MUO, metabolically unhealthy obesity; OR, odds ratio; CI, confidence interval. ^†^Multivariable model was adjusted for age (except for age subgroups), sex (except for sex subgroups), race and ethics, education level, smoking status (except for smoking status subgroups), alcohol consumption, physical activity (except for physical activity subgroups), daily water intake, CKD stage 3–5, and hyperuricemia.

In the sensitivity analyses (Supplementary Table S3), the associations between metabolic health-obesity phenotypes and kidney stones were largely unchanged when additionally adjusting blood pressures, glucose, lipid profiles, and HOMA-IR in the multivariate logistic regression model, or additionally adjusting important dietary factors in the model.

## Discussion

In this study, compared with metabolically healthy individuals with normal %BF, BMI, or WC, MHO individuals had significantly increased risk of kidney stones. The dose–response relationship between %BF and kidney stones was observed to be non-linear in metabolically healthy participants. The association between %BF and kidney stones was evident in different subgroups of sex, age, smoking status, and physical activity. These findings suggest that obesity assessed by body fat can independently contribute to kidney stones, even among individuals without metabolic abnormalities.

The association between obesity and kidney stones has been well established in many epidemiological studies (2, 3, 28, 29). However, a complex scenario has been proposed, in which obesity promotes both lithogenic process and insulin resistance, but obesity and insulin resistance promote a range of metabolic abnormalities, with the latter acting as determinants of kidney stones (28, 30). Therefore, it is difficult to determine whether obesity *per se* is causally involved in kidney stones disease or whether obesity and its related metabolic disorders jointly promote urinary stone formation. Assessing the risk of kidney stones among individuals cross-classified by metabolic health and obesity may help clarify the role of obesity in the development of kidney stones. In the present study, MHO individuals were not only free form metabolic disorders such as elevated blood pressure, elevated blood glucose, and dyslipidemia, but also were insulin sensitive. Even so, the risk of kidney stones was significantly higher in MHO individuals than in those with MHN, which validated the hypothesis that obesity can contribute to kidney stones formation even in the absence of metabolic abnormalities and insulin resistance.

To date, there are two studies explored the association between MHO (defined by BMI) and kidney stones (9, 10). Kim et al. found that among metabolically healthy individuals, the multivariable-adjusted HRs (95% CIs) for incident nephrolithiasis comparing BMI 25.0–29.9 kg/m^2^, and ≥30.0 kg/m^2^ with a BMI of 18.5–22.9 kg/m^2^ as the reference were 1.12 (1.03–1.22), and 1.72 (1.21–2.44), respectively, whereas the corresponding HRs (95% CIs) in metabolically unhealthy individuals were 1.27 (1.20–1.34), and 1.36 (1.22–1.51), respectively (9). Choi et al. found that compared to the MHN group, after adjusting for age and sex, the MHO group had an HR (95% CI) of 1.24 (1.18–1.31) for undergoing either extracorporeal shockwave lithotripsy or surgery during the follow-up, indicating that MHO individuals have a higher risk of developing symptomatic urolithiasis than their non-obese counterparts (10). Consistent with their results, in this nationally representative population, MHO group (defined by BMI) was a high-risk population for kidney stones. Moreover, we further found that the MHO phenotype defined by %BF and WC was also significantly associated with an increased risk of kidney stones. Of note, since %BF can well reflect obesity and is able to characterize obesity more accurately than BMI and WC, our findings provide convincing evidence to support that obesity *per se*, regardless of metabolic abnormalities or insulin resistance, is able to increase the risk of kidney stones. Hence, maintaining healthy body composition, normal weight, and WC are of great significance to prevent kidney stones for all adults. In addition, we found that the shape of the dose–response relationship between %BF and kidney stones was non-linear in metabolically healthy participants. Their relationship presents a rapidly increasing trend when the %BF elevated from 32 to 40%, suggesting that in this range of %BF, the risk of kidney stones increased more rapidly.

Although many questions remain on the link between obesity itself and kidney stones formation, several potential mechanisms may explain how obesity contribute to kidney stones disease in the absence of metabolic abnormalities. Obesity alters adipokine expression and has been shown to increase the levels of inflammatory cytokines, including tumor necrosis factor-α and interleukin-6 (31). Obesity has been associated with a decline in circulating plasma adiponectin, and this downregulation has been linked to increased oxidative stress and pro-inflammatory state (32, 33), which were both conditions promoting the formation of kidney stones. Furthermore, previous studies had also shown that obesity altered urine chemistry, correlated with lithogenic urinary risk factors, including uric acid supersaturation and lower urinary pH (34, 35), thereby increasing the risk of nephrolithiasis.

Kim et al. found that the association between obesity and incident nephrolithiasis was stronger in men (9). The reason for this sex difference is uncertain but may be related to the protective effect of estrogen against the formation of kidney stones (36). Compared with men with MHO, estrogen in MHO women may counteract the lithogenic effects of obesity. However, in the present study, no interaction was observed across subgroups of sex. The small sample size of our study makes it difficult to adequately test the association between subgroups, which may partially explain the differences of the results between ours and previous study.

This study found that the incidence of kidney stones in individuals with metabolically unhealthy status was higher than that in individuals with metabolically healthy status across all %BF categories. Because metabolic syndrome is a well-established risk factor for kidney stones (2), the results of ours are expected. This finding also re-emphasizes the clinical significance of the management of metabolic syndrome for the prevention of kidney stones.

The strengths of this study include using %BF and both BMI and WC to identify obesity, assessing the insulin resistance by HOMA-IR to better distinguish metabolically healthy individuals from those who are unhealthy, and comprehensively adjusting for confounders such as demographic, lifestyle, and comorbidities. However, this study also had several limitations. First, the assessment of kidney stones was based on self-report in NHANES, which may introduce a recall bias in our study. However, evidence shows that in a random sample of 60 self-reported kidney stones patients, medical records confirmed that 97% of them did have kidney stone diseases (37), which suggested that self-reported records were reliable for the outcome of kidney stones. Moreover, a previous study on kidney stones using NHANES data additionally considered a secondary outcome of self-reported stone passage that reflected symptomatic stones and found similar results regarding stone prevalence (38). Second, the causal association between MHO defined by %BF and kidney stones could not be evaluated due to the cross-sectional design. Further studies using a prospective cohort design will be helpful in understanding the causal relationship between MHO and nephrolithiasis. Third, information on the composition of kidney stones is unavailable in the NHANES, and the original records or imaging examination results of the types of stones (size, single/multiple, one side/both sides, staghorn or not) are also unavailable, hence we could not further explore the association between MHO and different types of kidney stones, which might help to provide valuable evidence on the pathophysiological mechanism of obesity itself on kidney stones formation. Finally, the NHANES only conducted DXA scans in participants aged 20–59 years, hence the participants in our study were mainly middle-aged adults. Since age differences exists in kidney stones prevalence (38), the results reported here may not necessarily generalize to populations of older adults.

In conclusion, in this nationally representative population in US, MHO phenotype defined by %BF exhibited an increased risk of kidney stones. Our findings indicate that regarding kidney stones prevention, all adults with obesity, even those with metabolically healthy status, should be encouraged to maintain a normal body composition through lifestyle interventions.

## Data availability statement

Publicly available datasets were analyzed in this study. This data can be found here: https://wwwn.cdc.gov/nchs/nhanes/continuousnhanes/default.aspx.

## Ethics statement

The studies involving human participants were reviewed and approved by the Centers for Disease Control and Prevention Research Ethics Review Board and written informed consent was obtained from all adult participants. The present analyses were approved by the Institutional Review Board of Peking university People’s Hospital (approval ID: 2020PHB 125–01). The patients/participants provided their written informed consent to participate in this study.

## Author contributions

WC, SM, TX, XH, and BW contributed to the conceptualization. SM, WC, and YH carried out the formal analysis. SM, WC, and LC involved in writing – original draft. GK, LX, QX, XH, and BW involved in writing – review and editing. XH and BW were responsible for resources. All authors contributed to the article and approved the submitted version.

## Conflict of interest

The authors declare that the research was conducted in the absence of any commercial or financial relationships that could be construed as a potential conflict of interest.

## Publisher’s note

All claims expressed in this article are solely those of the authors and do not necessarily represent those of their affiliated organizations, or those of the publisher, the editors and the reviewers. Any product that may be evaluated in this article, or claim that may be made by its manufacturer, is not guaranteed or endorsed by the publisher.
