# Supplementary material for: Association between metabolically healthy obesity and kidney stones: results from the 2011–2018 National Health and Nutrition Examination Survey
Source: Front Public Health. 2023 May 25;11:1103393. doi: 10.3389/fpubh.2023.1103393 (PMC10249726; doi:10.3389/fpubh.2023.1103393)
Supplement: Supplementary file 1 [file Table_1.docx]

Supplementary Table 1. %BF criteria used to determine participants’ obesity category

| **Category** | **Male** | |  | **Female** | |
| --- | --- | --- | --- | --- | --- |
|  | **20-39 years** | **40-59 years** |  | **20-39 years** | **40-59 years** |
| Underweight | < 8.0 | < 11.0 |  | < 21.0 | < 23.0 |
| Normal weight | 8.0-20.9 | 11.0-22.9 |  | 21.0-32.9 | 23.0-34.9 |
| Overweight | 21.0-25.9 | 23.0-28.9 |  | 33.0-38.9 | 35.0-40.9 |
| Obesity | > 26.0 | > 29.0 |  | > 39.0 | > 41.0 |
