# Supplementary material for: Association between metabolically healthy obesity and kidney stones: results from the 2011–2018 National Health and Nutrition Examination Survey
Source: Front Public Health. 2023 May 25;11:1103393. doi: 10.3389/fpubh.2023.1103393 (PMC10249726; doi:10.3389/fpubh.2023.1103393)
Supplement: Supplementary file 2 [file Table_2.docx]

**Supplementary Table 2. The association between metabolic health-obesity (defined by BMI or WC) phenotypes and kidney stones**

| **Metabolic health-obesity phenotypes** | **Using the BMI criteria** | | **Using the WC criteria**  **OR (95% CI)** ^†^ |
| --- | --- | --- | --- |
|  | **OR (95% CI)** ^†^ | |  |
| Metabolically healthy participants |  |  | |
| MHN | Reference | Reference | |
| MHOW | 2.20 (1.13-4.32) | 2.50 (0.53-11.88) | |
| MHO | 2.89 (1.01-8.24) | 3.46 (1.13-10.64) | |
| P for trend | 0.017 | 0.225 | |
| Metabolically unhealthy participants |  |  | |
| MUN | 1.72 (0.86-3.44) | 2.02 (0.57-7.18) | |
| MUOW | 2.72 (1.54-4.82) | 4.10 (1.13-14.84) | |
| MUO | 3.70 (2.06-6.64) | 5.31 (1.76-16.00) | |
| P for trend | <0.001 | <0.001 | |

BMI, body mass index; WC, waist circumference; MHN, metabolically healthy normal weight; MHOW, metabolically healthy overweight; MHO, metabolically healthy obesity; MUN, metabolically unhealthy normal weight; MUOW, metabolically unhealthy overweight; MUO, metabolically unhealthy obesity; OR, odds ratio; CI, confidence interval.

^†^ Multivariable model was adjusted for age, sex, race and ethics, education level, smoking status, alcohol consumption, physical activity, daily water intake, CKD stage 3-5, and hyperuricemia.
