# Supplementary material for: Association between metabolically healthy obesity and kidney stones: results from the 2011–2018 National Health and Nutrition Examination Survey
Source: Front Public Health. 2023 May 25;11:1103393. doi: 10.3389/fpubh.2023.1103393 (PMC10249726; doi:10.3389/fpubh.2023.1103393)
Supplement: Supplementary file 3 [file Table_3.docx]

**Supplementary Table 3. Sensitivity analyses of the association between metabolic health-obesity phenotypes (defined by %BF) and kidney stones**

| **Metabolic health-obesity phenotypes** | **Additionally adjusting blood pressures, glucose, lipid profiles, and HOMA-IR**  **(as continuous variables)** | | **Additionally adjusting important dietary factors, including dietary intake of protein, carbohydrate, fiber, and total fat**  **OR (95% CI)** ^‡^ |
| --- | --- | --- | --- |
|  | **OR (95% CI)** ^†^ | |  |
| Metabolically healthy participants |  |  | |
| MHN | Reference | Reference | |
| MHOW | 1.43 (0.48-4.23) | 1.46 (0.49-4.39) | |
| MHO | 2.64 (1.04-6.66) | 2.58 (1.01-6.63) | |
| Metabolically unhealthy participants |  |  | |
| MUN | 1.85 (0.74-4.67) | 1.99 (0.76-5.17) | |
| MUOW | 2.37 (1.06-5.33) | 2.64 (1.12-6.24) | |
| MUO | 2.81 (1.3-6.09) | 3.24 (1.46-7.18) | |

%BF, percent body fat; HOMA-IR, homeostasis model assessment of insulin resistance; MHN, metabolically healthy normal weight; MHOW, metabolically healthy overweight; MHO, metabolically healthy obesity; MUN, metabolically unhealthy normal weight; MUOW, metabolically unhealthy overweight; MUO, metabolically unhealthy obesity; OR, odds ratio; CI, confidence interval.

^†^ Multivariable model was adjusted for age, sex, race and ethics, education level, smoking status, alcohol consumption, physical activity, daily water intake, CKD stage 3-5, hyperuricemia, systolic blood pressure, diastolic blood pressure, glucose, triglycerides, high-density lipoprotein cholesterol, and homeostasis model assessment of insulin resistance.

^‡^ Multivariable model was adjusted for age, sex, race and ethics, education level, smoking status, alcohol consumption, physical activity, daily water intake, CKD stage 3-5, hyperuricemia, dietary protein intake, dietary carbohydrate intake, dietary fiber intake, and dietary intake of total fat.
